# Computed Tomographic Findings in Canine Otitis Externa

**DOI:** 10.1111/vru.70149

**Published:** 2026-03-06

**Authors:** Andrea Vila Cabaleiro, Tessa V. Procter, Anita Patel, Tobias Schwarz, Helen Dirrig, Yi Lin Tan

**Affiliations:** ^1^ Royal (Dick) School of Veterinary Studies University of Edinburgh, Roslin Edinburgh UK; ^2^ Department of Clinical Sciences and Services The Royal Veterinary College University of London Hertfordshire UK; ^3^ Veterinary Referral and Emergency Centre Godstone UK

**Keywords:** computed tomography, dogs, ear canal mineralization, ears

## Abstract

Otitis externa (OE) is a common condition in dogs. Otoscopic examination is the standard diagnostic procedure, but not all external ear canal structures can be assessed otoscopically. Computed tomography (CT) has become a gold standard in the assessment of middle ear disease. This study aimed to assess the validity of CT for canine OE and to compare CT‐specific and otoscopy‐specific findings that are consistent with OE. CT studies of dogs referred for suspected OE were blindly reviewed by three observers for ear canal wall mineralization, thickness, and contrast enhancement. Otoscopic findings were used as the reference standard. The sensitivity and specificity of CT for the diagnosis of OE were 98.2% (95% confidence interval [CI]: 90.4%–99.7%) and 60.0% (95% CI: 10.7%–76.6%), respectively. The positive predictive value (PPV) was 84.4%, and there was substantial agreement (weighted *κ* = 0.65) between CT and otoscopic diagnosis of OE. There was moderate agreement (weighted *κ* = 0.47) between the presence of CT ear canal wall contrast enhancement and the degree of erythema otoscopically, and moderate agreement (weighted *κ* = 0.58), comparing the consensus CT grade for ear canal wall thickness to the grade of ear canal stenosis on otoscopic examination. The presence of external ear canal wall mineralization on CT was independent of disease (*p* = 0.49) and disease duration (*p* = 0.26), indicating mineralization of the external ear canal wall on CT is not necessarily related to chronic OE. There is substantial agreement between CT and otoscopy, supporting its use as a diagnostic technique for OE.

**Abbreviations**
CT: computed tomography
HU: Hounsfield Units
OE: otitis externa
PPV: positive predictive value
QMHA: Queen Mother Hospital for Animals
ROI: region of interest
RVC: Royal Veterinary College
VREC: Veterinary Referral and Emergency Centre
WL: window level
WW: window width

## 1 Introduction

Computed tomography (CT) is a well‐established noninvasive diagnostic technique for the investigation of middle ear disease in dogs [1, 2, 3, 4]. Middle ear disease is often associated with otitis externa (OE) [5, 6, 7, 8], and a full evaluation of the external ear canal is important in the diagnostic work‐up of dogs with ear disease [4, 9, 10, 11]. The external ear canal is included in the field of view of CT examination of the middle ears, yet there is only very limited information published on CT external ear canal assessment in dogs [12] or humans [13, 14, 15]. Otoscopy is widely used to demonstrate and assess the ear canals in both acute and chronic disease, but it has limitations for which CT assessment could be of value, such as the evaluation of stenotic ear canals, tympanic membranes (TMs), and mineralization of the ear canal wall [9, 11, 16]. Ear canal wall mineralization is frequently seen on radiographs and CT in dogs and has been associated with chronic OE [4, 10, 17]. However, there are no evidence‐based studies confirming this, and ear canal mineralization is frequently seen in dogs without clinically manifest OE and may represent a degenerative process in dogs. The aims of this retrospective study were (1) to determine the sensitivity, specificity, and positive predictive value (PPV) of CT for the detection of OE; (2) to compare CT and otoscopy OE findings, assessing the agreement between the two diagnostic techniques; and (3) to correlate CT evidence of ear canal wall mineralization with the duration of OE. We hypothesized that (1) CT is a sensitive and specific modality for diagnosing OE with a high PPV; (2) there is good agreement between CT findings and otoscopic examination regarding ear canal thickening and stenosis, respectively; (3) the presence of ear canal wall contrast enhancement observed on CT does correlate with the presence of erythema assessed otoscopically; (4) the observation of TM on CT does correlate with TM visualization on otoscopy; and (5) OE duration and external ear canal wall mineralization are not associated.

## 2 Materials and Methods

### 2.1 Case Selection

This was a retrospective, multiple‐institutional observational study. Ethical approval was granted by the Clinical Research Ethical Review Board at the Royal Veterinary College (RVC) (URN: M2018 0149). The medical record database of the Veterinary Referral and Emergency Centre (VREC) in Surrey (UK) was searched to identify dogs with a reported history and clinical signs suggestive of OE. Key words used in the search included “otitis externa,” “stenosis,” “pruritus,” “ear exudate,” and “head shaking.” Dogs were included if they had undergone a dermatological work‐up consisting of a history of disease duration and an ear video‐otoscopy examination, in addition to a head CT scan, between January 2018 and February 2022. Inclusion was based on clinical suspicion of OE and the availability of both otoscopic and CT data, regardless of whether OE was ultimately confirmed in one or both ears. In addition, a second group of dogs with clinical signs suggestive of acute OE was identified from the database at the Queen Mother Hospital for Animals (QMHA), using the same inclusion criteria as above. For dogs with more than one CT scan during this period, only the first study (at the time of initial diagnosis) was included in the analysis. The following data were recorded: age, weight, gender, neuter status, breed, presenting clinical signs, duration of disease before referral, and otoscopic findings. Treatment and cytological results from external ear canal samples, along with culture and sensitivity results, were recorded (Supporting Information). The condition was defined as unilateral or bilateral based on clinical and video‐otoscopic examinations conducted by a board‐certified dermatologist, who recorded the affected ears. Final inclusion of cases was determined by an RVCS Recognized Specialist in Veterinary Dermatology (A.P.).

### 2.2 CT Technique and Examination

All dogs underwent a standardized head or aural CT with inclusion of the entire ear. All patients were positioned in standardized sternal recumbency under general anesthesia. Scans were performed using a 32‐slice helical multidetector scanner (Aquilion Lightning TSX‐035A, Toshiba America Medical Systems, Tustin, CA, USA) and a 320‐slice CT (Aquilion ONE–Genesis, Canon Medical Systems, Japan). Contrast medium was not administered in five dogs. In the remaining cases, contrast medium (2 mL/kg; Omnipaque, 300–350 mg iodine/mL, GE Healthcare, UK) was administered via the cephalic vein manually or using a pressure injector (Medrad Stellant CT Dual Injection System, Bayer Medical Care B.V., the Netherlands). Images were acquired in helical scan mode, at 120 kV and 35 (units) and 196 mAs tube settings, slide interval of 0.25–0.5 mm, and slice thickness of 0.5–1 mm in bone window and 1–2 mm in soft tissue window. A single post‐contrast phase was acquired 60 s after the initiation of contrast injection using a fixed acquisition delay for all patients.

### 2.3 Image Analysis

All images were reviewed on a workstation (Apple Mac Pro, Apple, USA) with a calibrated liquid crystal display (LCD) flat‐screen monitor using dedicated digital imaging and communications in medicine (DICOM) viewer software (Horos, Purview, Annapolis, MD, USA, version 3.3.6) independently and blindly by a diagnostic imaging intern (A.V.C.), a final‐year ECVDI diagnostic imaging resident at the time of data analysis (Y.L.T.), and an ECVDI‐certified radiologist (T.S.), with final diagnoses determined by consensus. All studies were randomized and anonymized prior to analysis. CT evaluations were performed in reconstructed transverse, dorsal, and sagittal planes, following multiplanar reconstruction, with the horizontal axis angled parallel to the palatine bone on the sagittal plane, vomer on the dorsal plane, and the vertical axis situated on the midline of the skull. Standardized bone (window level [WL] = 500, window width [WW] = 2500) and pre‐ and post‐contrast soft tissue windows (WL = 35, WW = 350) were used for evaluation of the images.

**FIGURE 1:**
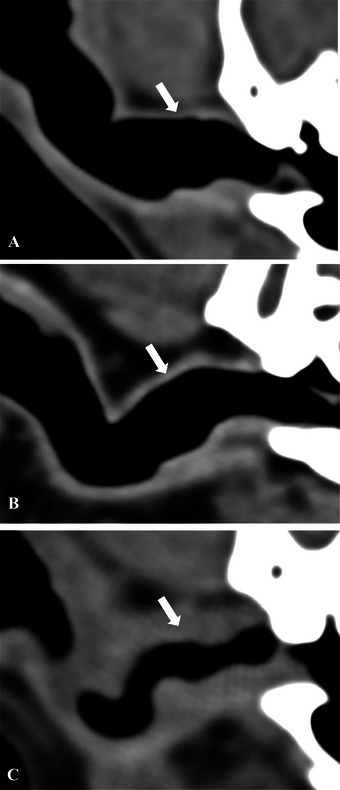
Pre‐contrast transverse CT images, in standardized soft tissue window, showing normal (A), mildly thickened (B), and markedly thickened horizontal ear canal (C). Subjective assessment along the dorsal wall at 0.5 length of the *pars horizontalis* at the level of the maximal ear canal diameter (arrows).

The left and right external ear canals and associated structures were assessed separately but not blindly. For each external ear, several assessments were recorded from the transverse plane images. First, ear canal wall thickness was evaluated using the pre‐contrast soft tissue reconstruction along the dorsal wall at 0.5 length of the *pars horizontalis* at the level of maximal ear canal diameter (Figure 1) and subjectively graded as (0) normal, (1) mildly thickened, or (2) markedly thickened.

Second, the presence or absence of contrast enhancement in the horizontal ear canal wall was evaluated using pre‐ and post‐contrast soft tissue reconstruction in the transverse plane. A manually drawn region of interest (ROI) was used to measure x‐ray attenuation in Hounsfield Units (HU) at the same location, along the dorsal wall at 0.5 length of the *pars horizontalis*, at the level of the maximal ear canal diameter. The presence of wall contrast enhancement was confirmed if there was 25% increase in HU between pre‐ and post‐contrast series.

**FIGURE 2:**
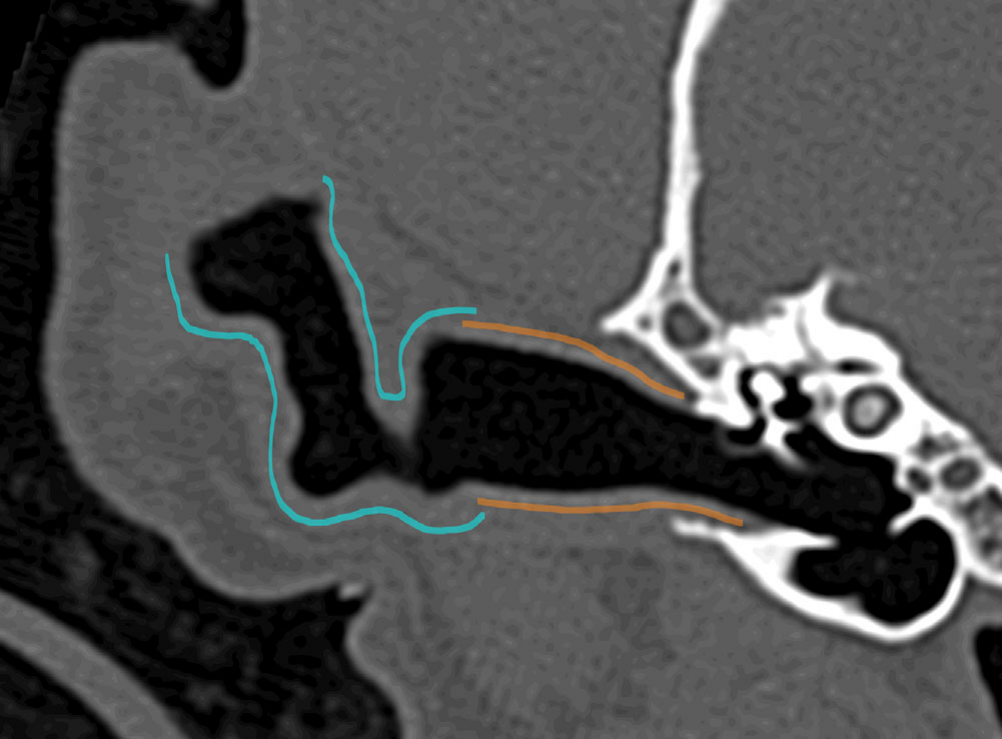
Transverse CT image in standardized bone window of the ear canal of a 6‐year‐old neutered male Cockapoo, showing the regions of the annular cartilage (orange outline) and auricular cartilage (blue outline). The annular cartilage typically extends from the tympanic membrane to approximately the lateral 1/3 of the *pars horizontalis*. The auricular cartilage typically extends from this point to the free margin of the pinna (not shown in this image). Cartilage anatomy may vary between breeds.

Third, ear canal wall mineralization was evaluated using standardized bone reconstruction in the transverse plane and graded on a 3‐point scale as (0) absent, (1) with wall mineralization ≤25%, and (2) >25% of the entire ear canal using bone algorithm images. Additionally, the location of the ear canal mineralization, if present, was scored as being in (0) the annular cartilage, (1) the auricular cartilage, or (2) annular and auricular cartilages (Figure 2).

Finally, the TM was assessed using standardized bone reconstruction in the transverse plane on a 3‐point scale as (0) not visible, (1) directly visualized, and (2) indirectly visualized. Direct visualization was defined as clear identification of the TM, whereas indirect visualization referred to cases where the TM was not distinctly seen but inferred from adjacent tissue.

For the purposes of this study, a diagnosis of OE on CT was made when either ear canal wall thickening or contrast enhancement was present. Ears that showed only ear canal wall mineralization, without adjacent thickening or contrast enhancement, were not considered indicative of OE and were classified as disease‐free cases.

### 2.4 Otoscopy and Ear Flushing

Video‐otoscopy (Karl Storz 624325 20 and Karl Storz Xenon Nova 175 light 201315 20; Tuttlingen, Germany) was performed on all ears by a board‐certified dermatologist (A.P. and the QMHA dermatologist team) on the same day after the CT examination. For each external ear, otoscopic findings were graded on the presence/absence of erythema and stenosis using a 3‐point scale as (0) absent, (1) mild, or (2) marked. Moreover, the visibility of the TM was assessed.

Ears were classified otoscopically as either disease‐free or diseased (OE), based on otoscopic evidence of ear canal stenosis and/or erythema. Cytology of the ear discharge was considered (90% cases) where available. Diseased ears were further subdivided into acute (≤4 weeks duration prior to presentation at the referral center) and chronic (>4 weeks).

Most chronic OE (77.8%) cases were treated with oral and/or topical glucocorticoids for 7–14 days prior to imaging to reduce edema and stenosis of the external ear canals. During otoscopy, cytological samples from the ear canals were collected prior to ear flushing, followed by examination of the ear canal and, when possible, of the TM. All video otoscopic images were recorded and reviewed by board‐certified dermatologists.

The grade of thickness of the ear canal wall and the presence of horizontal ear canal wall contrast enhancement evaluated tomographically were compared with the degree of stenosis and the presence of erythema of the ear canal wall evaluated otoscopically, respectively, using statistical analysis.

Ear canal wall mineralization evaluated tomographically was compared with the duration of the ear disease. Assessment of the TM by CT was compared to otoscopy findings.

### 2.5 Statistical Analysis

Statistical analysis was performed by a diagnostic imaging resident (T.V.P.) who had statistical training as part of her PhD, using open‐source software (R v4.2.1, RStudio Team 2022, RStudio: Integrated Development Environment for R: RStudio, PBC) and RStudio v2022.12.0.353 (R Core Team 2022, R: A Language and Environment for Statistical Computing: R Foundation for Statistical Computing). Statistical significance was defined as *p* value <0.05. The PPV of CT for diagnosing OE and the overall sensitivity and specificity of CT for the diagnosis of OE compared to the reference standard (dermatological examination and direct otoscopy findings) were calculated. The agreement between CT ear canal wall contrast enhancement and the degree of wall thickening, the presence of otoscopically determined ear canal erythema, and the degree of stenosis was assessed using Cohen's kappa. Fisher's exact test was used for the assessment of ear canal wall mineralization evaluated on CT and disease duration.

## 3 Results

### 3.1 Population

In total, 71 dogs referred for investigation of OE were retrieved from the VREC database from January 2018 to January 2022, and 36 dogs referred for acute OE investigation from the QMHA imaging database. Of these, 40 dogs met the inclusion criteria, with a total of 80 ears evaluated. The study population consisted of 18 neutered males, 9 entire males, 10 neutered females, and 3 entire female dogs. A variety of different breeds were identified, with the most common being French Bulldogs (3/40), Cocker Spaniels (7/40), Cockapoos (3/40), Labrador Retrievers (3/40), Labradoodles (2/40), other mixed‐breed dogs (2/40), and one of the following breeds: Welsh Springer Spaniel, Brussels Griffon, Sheepdog, Fox Terrier, English Setter, Flat‐coated Retriever, English Springer Spaniel, Sprocker, Beagle, Toy Poodle, Tibetan Terrier, Cavachon, Scottish Terrier, Havanese, Shar‐Pei, Parson Russell Terrier, West Highland White Terrier (WHWT), Italian Spinone, Kooikerhondje, and Cavalier King Charles Spaniel. The mean body weight was 16.27 kg (range: 5.8–41.2 kg). The mean age was 6.1 years (range 1.4–14.0 years). At the time of presentation, 12/40 dogs (30%) had identifiable comorbidities: eight dogs with atopy, three dogs with pyoderma, and one with hypothyroidism. Table 1 highlights the number of chronic and acute OE cases, as well as disease‐free cases.

**TABLE 1:** The presence or absence of otitis externa (OE) and, when present, categorizes cases as acute (≤4 weeks) or chronic (>4 weeks) based on the duration of symptoms prior to referral in 80 ears.

Duration of symptoms prior to referral	Number (%)
>4 weeks (chronic cases)	45 (56.2)
≤4 weeks (acute cases)	10 (12.5)
Disease‐free	25 (31.3)

### 3.2 Otoscopic and Dermatological Findings

OE was identified in 55/80 ears (68.8%), with bilateral involvement in 18/40 dogs (45.0%) and unilateral involvement in 19/40 dogs (47.5%). The remaining 3/40 dogs (7.5%) showed no evidence of OE in either ear. Among the 25/80 ears (31.2%) without evidence of OE, otoscopic findings were also unremarkable, with no signs of erythema or stenosis. Ear canal stenosis was reported in all dogs with OE (53/53 ears; data on ear canal stenosis were unavailable in 2 ears); 12/53 were graded as mild, and 41/53 were graded as marked. Erythema of the horizontal ear canal was observed in 49/51 ears with OE (96.1%), whereas data were missing for 4 ears. Additionally, TM was visualized in 25/80 ears (31.3%) during the otoscopic examination. In all ears classified as affected by OE (55/55), at least one otoscopic abnormality diagnostic of OE (ear canal stenosis and/or erythema) was present; specifically, the two ears with missing stenosis data exhibited erythema, and the four ears with missing erythema data exhibited stenosis. Additionally, TM was visualized in 25/80 ears (31.3%) during the otoscopic examination.

### 3.3 Imaging Findings

During CT evaluation, 64/80 ears (80.0%) were diagnosed with OE by the radiologists. Among these, 5 dogs did not receive IV contrast or had no available data regarding contrast administration; therefore, contrast enhancement could only be assessed in the remaining 54 ears. Ear canal wall contrast enhancement was present in 36/54 ears (66.7%). Ear canal wall thickening was noted in 58/64 ears (90.6%), with 22/58 graded as mild and 36/58 as marked. Of the 40 dogs, 27 (67.5%) were diagnosed with bilateral OE and 10 (25.0%) with unilateral OE based only on CT evaluation.

In 63/80 ears (78.8%), ear canal wall mineralization was noted: 50/63 ears had ≤25%, and in 13/63 ears, >25% of the canal was affected. The annular cartilage was affected in 31/63 ears (49.2%), the auricular cartilage in 8/63 ears (12.7%), and both in 24/63 ears (38.0%).

Of the 55 ears diagnosed with OE on dermatological examination, 54 ears had a CT diagnosis of OE, giving a sensitivity of 98.2% (95% confidence interval [CI]: 90.4%–99.7%). Of the 25 ears without evidence of OE on dermatological examination, 15 were identified as disease‐free on CT, giving a specificity of 60.0% (95% CI: 10.7%–76.6%). The PPV of CT for diagnosing OE in this population was 84.8%.

**FIGURE 3:**
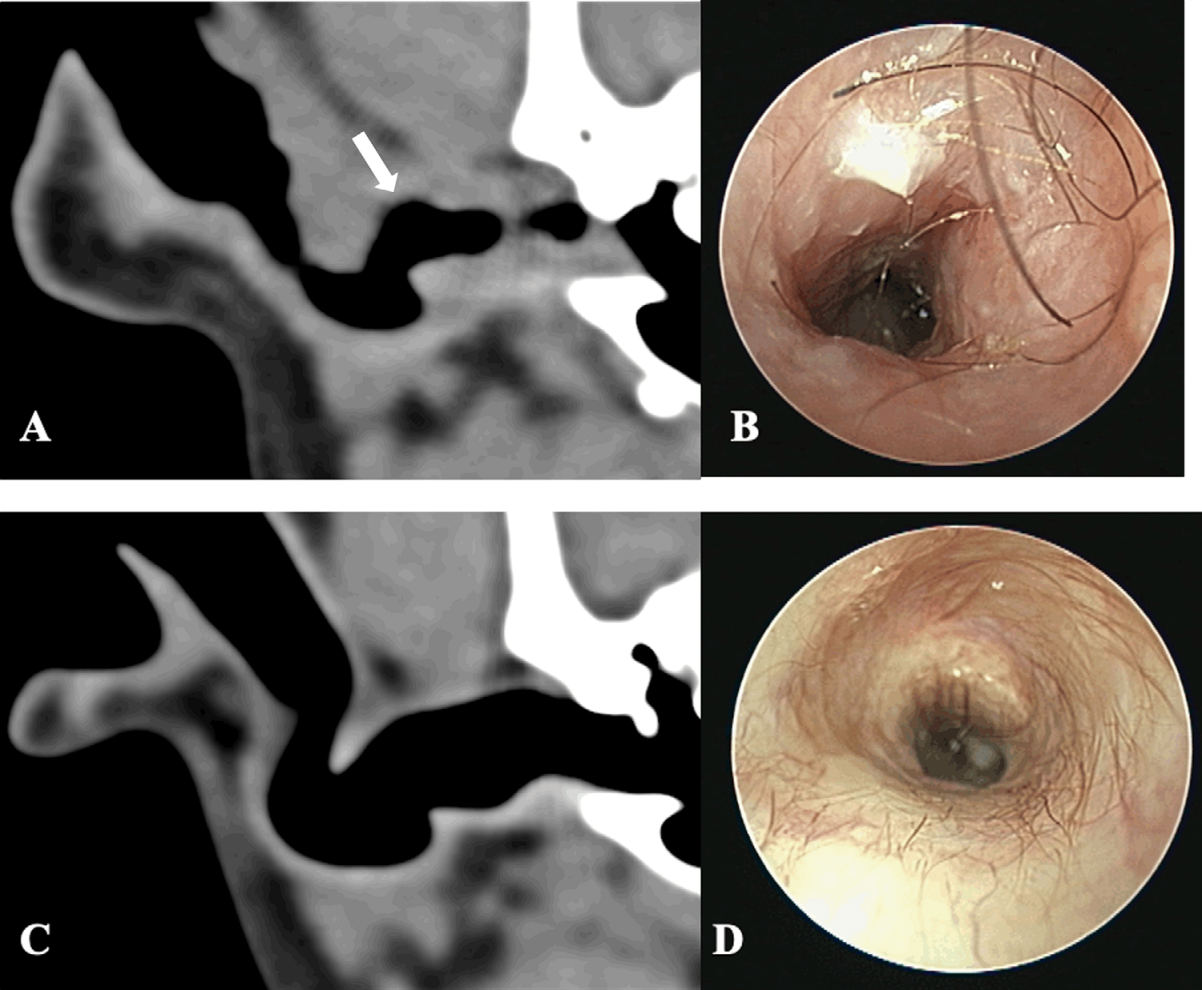
Transverse CT images, pre‐contrast standardized soft tissue window of the ear canal of two dogs (A and C) and otoscopy images after ear flush of the same dogs (B and D). Marked thickening of the horizontal part of the right ear canal wall (arrow) in an 8‐year‐old neutered female Cockerpoo (A). Otoscopy images of the same patient with marked stenosis, as a triangular shape in the lumen of the ear canal is noted (B). Absence of horizontal ear canal wall thickening of the right ear of a 4‐year‐old entire female Fox terrier (C). Otoscopy images of the same patient without the presence of stenosis (D).

There was moderate agreement between CT ear canal wall thickness grade and otoscopic ear canal stenosis grade (weighted *κ* = 0.58) (Figure 3). Otoscopic assessment for ear canal stenosis was not available for two ears.

**FIGURE 4:**
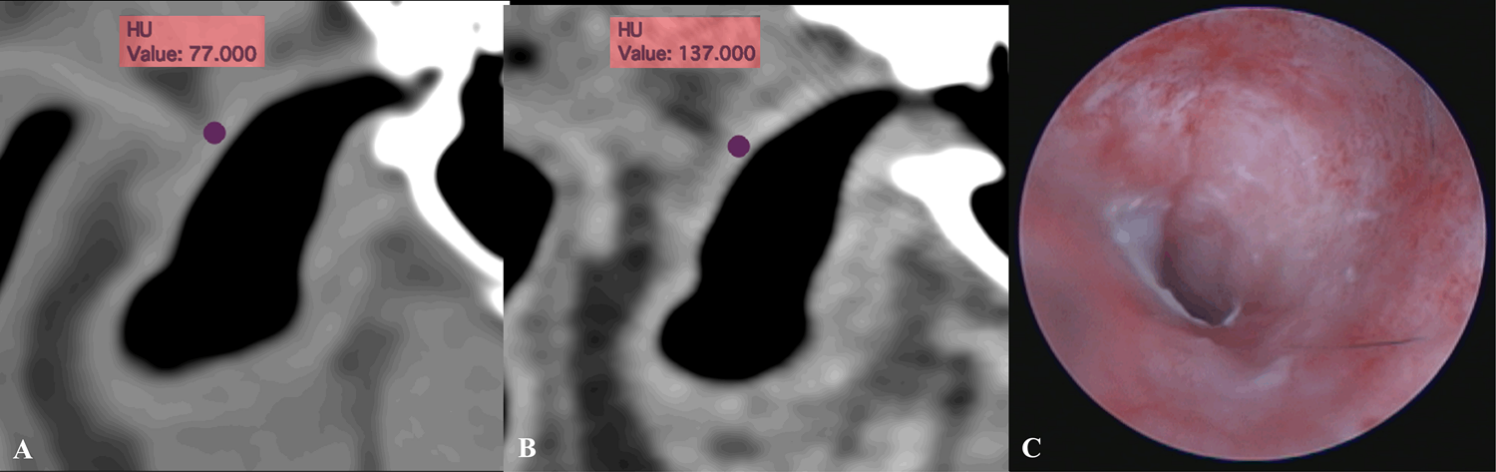
Transverse CT images of the right horizontal ear canal of a 9‐year‐old neutered male Cocker Spaniel, using soft tissue window, show pre‐contrast attenuation of HU = 77 (A) and post‐contrast attenuation of HU = 137 (B). These findings indicate contrast enhancement of the ear canal wall (oval ROI). Otoscopy image of the right ear of the same dog exhibits marked erythema (C).

There was moderate agreement between CT ear canal wall contrast enhancement and the presence of erythema otoscopically (weighted *κ* = 0.48). Of the 49 ears with erythema, 10 could not be compared with CT ear canal wall contrast enhancement due to the absence of post‐contrast images. Therefore, among the remaining 39 ears with erythema on otoscopic examination, 28 (70%) had ear canal wall contrast enhancement (Figure 4). Of these 28 ears, 3 had an acute presentation, and the remainder were chronic. The correlation between CT findings related to OE and otoscopic diagnosis of OE is shown in Table 2.

**TABLE 2:** Correlation of CT findings with otoscopic diagnosis of otitis externa (OE) in 80 ears.

CT finding	Otoscopic OE ears (*n* = 55)	Otoscopic disease‐free ears (*n* = 25)
**Ear canal wall thickening**	51 (92.7%)	7 (28.0%)
**Ear canal contrast enhancement** ^a^	30/45 (66.7%)	6/25 (24.0%)
**CT signs of OE (defined as ear canal wall thickening and/or contrast enhancement)**	54 (98.2%)	10 (40.0%)
**No CT signs of OE**	1 (1.8%)	15 (60.0%)

Abbreviation: CT, computed tomography; TM, tympanic membrane.

^a^ CT contrast enhancement data were unavailable in 10 ears, all diagnosed with OE on otoscopy; therefore, contrast enhancement percentages are calculated only from ears with available post‐contrast CT images. These 10 ears exhibited ear canal wall thickening on CT and were therefore diagnosed with OE on CT based on wall thickening alone.

**FIGURE 5:**
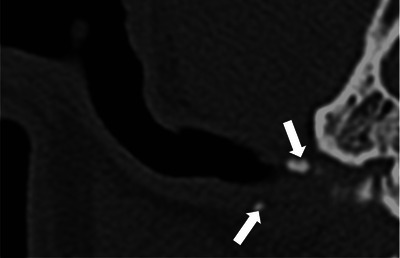
Transverse CT image in a standardized bone window showing the ear canal wall with ≤25% mineralization of the annular cartilage (arrows) in a 2‐year‐old entire male French Bulldog, 7 days after the onset of clinical signs of OE.

In 63/80 ears (78.75%), wall mineralization was observed on CT. The extent of mineralization in disease‐free ears (20/25) was ≤25% in 17/20 and >25% in 3/20 ears; in chronic OE (35/45), it was ≤25% in 25/35 and >25% in 10/35 ears, and in acute OE (8/10), it was ≤25% in 8/8 and >25% in 0/8 ears. When assessing the presence of mineralization and disease duration, mineralization was seen in 80% (20/25) of disease‐free, 77.8% (35/45) of chronic, and 80% (8/10) of acute OE. There was no significant association between the presence of OE and its duration (acute or chronic) with the presence of ear canal wall mineralization on CT (Fisher's exact test, *p* = 0.49 and *p* = 0.26, respectively) (Figure 5).

**FIGURE 6:**
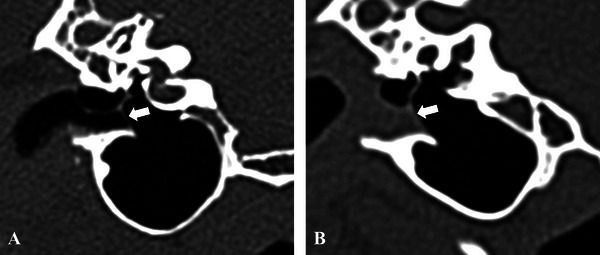
Transverse images in standardized bone window showing direct visualization (TM clearly visualized) of the TM in a 6‐year‐old neutered male Labradoodle (A) and indirect visualization (TM not directly resolved but inferred from adjacent tissue) in a 6‐year‐old neutered male Cockapoo (B). It is important to note that CT identification of the TM does not confirm its structural integrity (i.e., whether it is intact or ruptured).

Visualization of the TM was achieved in 25/80 ears (31.3%) during otoscopy. In contrast, CT allowed its visualization in 68/80 ears (85.0%), with direct visualization in 27 of those 68 ears and indirect visualization in 41 (Figure 6 and Table 3).

**TABLE 3:** Comparison of tympanic membrane (TM) visibility in 80 ears by otoscopic examination and CT.

TM assessment	Otoscopy (*n*, %)	CT direct visualization (*n*, %)	CT indirect visualization (*n*, %)	CT not visible (*n*, %)
**Visible TM**	25/80 (31.3)	27/68 (39.7)	41/68 (60.3)	12/80 (15)

*Note*: Computed tomography visibility is classified further as direct (TM clearly visualized), indirect (TM not directly resolved but inferred from adjacent tissue), and not visible. Direct and indirect CT percentages are calculated out of the 68 ears where the TM was visible on CT.

## 4 Discussion

This is the first study describing detailed CT findings of canine OE with otoscopic validation.

The findings of this study support our first hypothesis that CT is a sensitive modality for diagnosing OE, with a high PPV of 84.4%. However, this result should be interpreted with caution, as the high PPV is likely influenced by the high prevalence of OE in our study population, which consisted predominantly of dogs referred for suspected OE. This referral bias may not reflect the general dog population, and CT's predictive value in a broader clinical setting may be lower. Future studies in more diverse populations are warranted. Conversely, our hypothesis that CT would be a specific modality for diagnosing OE was not supported, and specificity was low/moderate (60.0%), with a wide CI. This broad interval likely reflects the limited number of disease‐free cases in our sample, which reduces the precision of the specificity estimate and limits its generalizability. On dermatological examination, 25 ears were classified as disease‐free; only 15/25 were also classified as disease‐free on CT. The discrepancy was due to 10/25 ears assessed as having wall thickening (7/10) and contrast enhancement of the horizontal ear canal (6/10). A possible explanation for the poor specificity of CT could be the absence or reduced amount of peri‐aural fat, which brings neighboring structures, such as the temporalis muscle, parotid gland, and lymph nodes, closer to the horizontal ear canal [18, 19]. This proximity complicates the accurate delineation of the ear canal's boundaries on CT and may give the false impression of mural thickening. Another factor may be normal anatomic variations, such as increased ear canal vascularization and folding of the horizontal ear canal, which can resemble ear canal wall thickening in certain breeds, such as brachycephalics, due to their skull conformation [20, 21, 22]. Lastly, the small number of disease‐free ears in our study may limit the accuracy of the specificity.

Our hypothesis that the degree of mural thickening on CT would correlate with the degree of ear canal stenosis on otoscopy was supported, with moderate agreement (weighted *κ* = 0.58) between the two diagnostic techniques. CT findings and otoscopic assessments agreed in the majority of cases (73.1%), suggesting that CT may be a useful tool for estimating external ear canal stenosis based on ear canal wall thickening. In contrast, a recent feline study reported poor agreement between CT ear canal wall thickening and stenosis on otoscopy, likely due to small sample size and the small size of the feline ear canal, making otoscopic examination more challenging [23]. Variations in ear canal anatomy may also contribute to differing interpretations of wall thickening or stenosis [5, 20, 21, 22]. Therefore, being aware of these variations is crucial for accurate interpretation.

A correlation between the presence of ear canal wall contrast enhancement observed on CT and the presence of erythema assessed otoscopically was demonstrated, showing moderate agreement (weighted *κ* = 0.47). These findings suggest that these diagnostic techniques may provide consistent assessments of external ear canal inflammation. It is important to note that erythema signals a superficial abnormality, whereas contrast uptake on CT reflects a full‐thickness or deeper tissue change. Although superficial findings likely commonly correspond to deeper pathology, this may not always be the case, and this distinction should be considered when comparing otoscopy and CT results. In contrast, a recent feline study reported poor agreement between video‐otoscopy and CT [23]. This feline study suggests that CT may offer greater sensitivity in detecting inflammation, particularly when otoscopy findings are inconclusive or affected by factors, such as prior anti‐inflammatory medication use or pigmentation. Furthermore, the lack of correlation in that feline study may be due to a smaller sample size and the fact that both video‐otoscopy and head CT were performed within a 3‐week period, rather than on the same day as in our study, where video‐otoscopy was performed immediately after the CT examination. One limitation of our study was the assessment of ear canal contrast enhancement only in the horizontal ear canal rather than in both vertical and horizontal as during otoscopic examination [24]. This approach was adopted to simplify the study protocol and promote interpreter accuracy. Future research should consider assessing contrast enhancement in multiple sites to provide a more comprehensive evaluation.

Our hypothesis that the observation of the TM on CT correlates with otoscopy was not fully supported. The low number of TMs detected by video‐otoscopy is thought to be due to ear canal edema and/or stenosis or breed‐related anatomical variations, particularly observed in brachycephalic breeds and Cocker Spaniels [25, 26, 27, 28]. In our study, the TM was visualized on CT in 85% of ears, which is higher than the 25% reported in another recent study [29]. This discrepancy may be partly due to our inclusion of both direct and indirect visualization, with a higher proportion of cases falling under indirect visualization (60.3% vs. 39.7%). These cases involved TM inference based on surrounding soft tissue, which can lead to overestimation. We used a standardized bone window in transverse planes, whereas Alves‐Nores et al. [29] emphasized the use of lung windows and dorsal reconstructions. However, the authors of that study also stated that, in their opinion, the optimal plane to identify the TM is the transverse one [29]. These technical differences may account for the discrepancies in visualization rates between our study and theirs. We acknowledge the risk of misinterpreting soft tissue outlines as intact TMs, especially in effaced or ruptured cases. Importantly, identification of the TM on CT does not confirm its integrity, as perforations may not be reliably detected. Stokowski et al. demonstrated low diagnostic accuracy and fair interobserver agreement for identifying TM perforations on CT, highlighting the potential for both false‐positive and false‐negative interpretations [30]. Therefore, we recommend that CT findings be interpreted with caution and confirmed by otoscopy when possible, to avoid overreliance on imaging alone. The referenced study describes positive contrast CT canalography as a safe and complementary method for assessing TM integrity in dogs, potentially offering higher sensitivity than otoscopic examination for diagnosing TM rupture [29].

Our hypothesis that ear canal wall mineralization would not correlate with disease was supported (*p* = 0.49). Additionally, the presence of external ear canal mineralization was not associated with disease duration (*p* = 0.26), with mineralization commonly seen in disease‐free (80%) and acute OE (80%) ears. There is no appreciable trend between disease, chronicity, and mineralization. Anecdotally, mineralization has often been used as a marker of chronicity and the presence of ear disease [5, 12]. Due to the limited number of acute cases in our study, one hypothesis is that ear canal mineralization might develop subclinically. Additionally, some of these cases might not truly represent acute episodes given the referral center setting; it is possible that these patients experienced prior episodes of OE that were not documented in their history or observed by the owner. Similarly, the so‐called disease‐free ears may have had prior episodes, suggesting that the underlying process could be chronic rather than purely acute. Consequently, further investigations involving a larger cohort of patients with acute OE could provide valuable insights. Alternatively, it is conceivable that mineralization could be considered a normal degenerative process within certain canine populations, a hypothesis that also requires further investigation according to Foster et al. [5]. Interestingly, in our canine population, the annular cartilage was the most frequently affected. This may be potentially attributed to anatomical factors and mechanical stress. Positioned at the base of the ear, the annular cartilage bears the weight of the ear, subjecting it to increased pressure, which may contribute to its heightened vulnerability to mineralization.

In our study, French Bulldogs and Cocker Spaniels were slightly overrepresented breeds undergoing CT and otoscopic examinations for OE, aligning with previous reports [20, 21, 22, 28, 31, 32]. This may be due to a higher prevalence of underlying dermatological or systemic conditions, including allergic disease (e.g., atopy), endocrine disorders, and keratinization defects, as well as the specific ear canal and pinna anatomy in these breeds [7, 9, 20, 33, 34, 35, 36].

In our study, some other limitations emerged: its retrospective design and the relatively small sample size, particularly of disease‐free and acute cases. OE is frequently managed with medical treatment in general practice, often without advanced diagnostic imaging. Within the referral context, only a subset of cases suspected of OE underwent CT examinations, whereas others, presenting with peripheral vestibular signs potentially linked with ear infections, were directed toward MRI investigations. This approach contributed to the modest number of patients included in this study. Furthermore, the population was relatively heterogeneous, as evidenced by the variety of breeds represented. Furthermore, ear canal thickness was graded subjectively rather than through objective measurement. This decision was made due to the significant breed‐ and size‐related anatomical variability in dogs, which complicates establishing standardized measurement criteria. Although subjective grading may limit reproducibility, it provided a practical approach for consistent assessment across our diverse population. Additionally, a single post‐contrast CT acquisition was performed using a fixed delay of 60 s for all patients, rather than tailoring scan timing to individual physiological parameters. As a result, some dogs may have been imaged during a true venous phase while others during a delayed phase, which may have led to under‐ or overestimation of contrast enhancement. Another limitation is that, although this study observed superior utility of CT over otoscopic examination for TM identification, no statistical analysis was performed to assess significance, as it was not the primary aim of our study. Further research may be needed for greater clarity on this topic. Lastly, although the observers were aware that the dogs were referred for suspected OE, potentially introducing observer bias, they were blinded to the otoscopic findings. This awareness may have increased the likelihood of interpreting CT scans as abnormal, potentially inflating the sensitivity and reducing the specificity of CT for detecting OE. This form of bias is difficult to avoid in a referral setting, where the majority of cases present with clinical signs suggestive of ear disease. Consequently, the inclusion of truly normal dogs was limited. Nonetheless, this case distribution reflects the nature of most clinical and otoscopic studies in referral populations and the typical patient spectrum encountered in such settings.

In conclusion, this is the first study describing detailed CT findings of canine OE with otoscopic validation. The results demonstrate a correlation between the degree of ear canal wall thickness and the presence of contrast enhancement on CT examination, with the degree of stenosis and presence of erythema observed on otoscopic examination, respectively. CT is a sensitive diagnostic adjunct to otoscopic examination for canine OE. Contrary to our expectations, our study accentuates the potential superior utility of CT over otoscopic examination for the identification of the TM. Care should be taken when interpreting the integrity of the TM. Interestingly, our findings indicate that there is no relationship between duration (acute or chronic) and presence of OE with external ear canal wall mineralization, suggesting that a degenerative process should also be considered.

## Author Contributions

Conception and design: Andrea Vila Cabaleiro, Yi Lin Tan, Anita Patel, Tobias Schwarz, and Helen Dirrig. Acquisition of data: Andrea Vila Cabaleiro, Yi Lin Tan, and Anita Patel. Analysis and interpretation of data: Andrea Vila Cabaleiro, Yi Lin Tan, Tessa V. Procter, and Tobias Schwarz. Drafting the article: Andrea Vila Cabaleiro, Yi Lin Tan, and Tobias Schwarz. Revising article for intellectual content: Andrea Vila Cabaleiro, Yi Lin Tan, Anita Patel, Tobias Schwarz, Tessa V. Procter, and Helen Dirrig. Final approval of the completed article: Andrea Vila Cabaleiro, Yi Lin Tan, Anita Patel, Tobias Schwarz, Tessa V. Procter, and Helen Dirrig. Agreement to be accountable for all aspects of the work in ensuring that questions related to the accuracy or integrity of any part of the work are appropriately investigated and resolved: Andrea Vila Cabaleiro, Yi Lin Tan, Anita Patel, Tobias Schwarz, Tessa V. Procter, and Helen Dirrig.

## Disclosure

An EQUATOR network was used. This research was accepted as oral presentations at the EVDI Conference in Edinburgh, UK, on September 17, 2022 (proceedings, p. 105) and at the EVDI Conference in Athens, Greece, on September 19, 2024 (proceedings, p. 72).

## Conflicts of Interest

The authors declare no conflicts of interest.

## Supporting information




**Table S1**: Clinical signs for the 37 out of 40 dogs diagnosed with bilateral or unilateral otitis externa.

## Data Availability

Public data repository with shared folder in a file hosting service (Dropbox).
