# Supplementary material for: Computed Tomographic Findings in Canine Otitis Externa
Source: Vet Radiol Ultrasound. 2026 Mar 6;67(2):e70149. doi: 10.1111/vru.70149 (PMC12964102; doi:10.1111/vru.70149)
Supplement: Supplementary file 1 — Table S1: Clinical signs for the 37 out of 40 dogs diagnosed with bilateral or unilateral otitis externa. [file VRU-67-0-s001.docx]

**Supplementary material**

| Presenting clinical signs in 37 dogs | Number (%) |
| --- | --- |
| Head shaking | 35 (94.6) |
| Malodour | 32 (86.5) |
| Pruritus | 14 (37.8) |
| Erythema and oedema of the pinna | 11 (29.7) |
| Discharge | 18 (48.7) |
| Neurological signs (head tilt, deafness, Horner’s syndrome and loss of balance) | 7 (18.9) |

Table 1 Clinical signs for the 37 out of 40 dogs diagnosed with bilateral or unilateral otitis externa.

Treatment and cytological results from external ear canal samples, along with culture and sensitivity (C&S) results, were recorded.

*Cytological findings and bacterial and culture sensitivity testing*

Samples for cytological evaluation and bacterial C&S were obtained either at the time of CT and video otoscopic examination and ear flushing or at the initial examination, typically 10–15 days before the CT scan.

Over half of the ears, 49/80 ears (61.3%), were affected with bacteria (rod-shaped, coccoid or a combination of both) and *Malassezia* yeast. Coccoid bacteria were present in 13/49 ears (26.5%), rod-shaped bacteria in 13/49 (26.5%), a combination of the two in 7/49 (14.2%) and *Malassezia in* 30/49 (61.2%)*.* An infectious agent was not found in 23/80 ears (28.8%). There was no data available of the remain 8/80 ears (10.0%). Bacterial C&S testing was performed on 28/49 ears affected with bacteria in the cytology. All the rod-shaped bacteria identified were *Pseudomonas aeruginosa,* and the coccoid bacteria primarily identified were *Staphylococcus pseudointermedius* and *Streptococcus canis*, with one case of *Escherichia coli*.

*Treatment*

Most of the dogs received medical treatment alone (65/80, 81.25%) for the OE. The remaining dogs (15/80, 18.75%) received surgical treatment in at least one of the ears. The selection criteria for the surgical treatment in our study was in most of the dogs due to chronic recurrent external ear canal infection and/or inflammation, marked stenosis and concomitant otitis media.
